# Nutrition

**Published:** 1877-03

**Authors:** A. L. Browne


### NUTRITION.

BY A. L. BROWNE.

*A Thesis presented to the Faculty of the Ohio College of Dental Surgery for the degree of D. D. S.*

The subject of nutrition considered in the widest acceptation of that term includes that whole series of operations, which prepare the alimentary materials to be appropriated by the different tissues of the organism, and secondly, their conversion into organized tissues. But in a more limited sense it is restricted to the latter of these operations that of histogenesis or tissue-formation, The blood carries the nutritive materials to all parts of the body. These materials must be in a liquid condition, and are brought into that condition by the digestive process. Fat forms an exception, it being taken into the blood in the form of an emulsion. Thorough aeration of the blood is indispensable to healthy histogenesis.

The exercise of the function of nutrition would necessarily involve an impoverishment of the blood, were it not for certain organs that supply the blood with nutritive materials, by reducing them to a liquid condition, the state in which they are taken into the blood, thus helping it in the requisite state of purity. Each tissue of every organ possesses a capacity for growth and development, in virtue of which it passes under the influence of the steady heat, through a series of successive phases. Thus we perceive that there is an intimate relation between nutrition and animal heat. Carpenter claims that this capacity for growth and development is derived from an original germ, but that it diminishes with the advance of life until the power of maintenance is no longer equal to the disintegration of the system. Notwithstanding the diversity in the structure and composition of the various tissues, the blood supplies them all with the materials they require. Each tissue may be said to possess an "elective affinity," by which power it is enabled to select from the blood the proper principles necessary for their development, growth, and maintenance. It has been shown that the formative capacity does not exist in the tissues alone, but that it is also shared by the blood, for there are certain simple kinds of tissue, which appear to take their origin directly in it. Others which can not thus be said to take their origin, may be said to have their development controlled by the amount of their special pabulum the blood may contain. Thus, adipose tissues are formed under certain conditions, when there is a large amount of fat in the blood. An increase in iron produces an increase in the red corpuscles of the blood. If one of any pair of secreting organs be removed, the other will increase in size in order to make up the loss in secreting power. This is true of the kidneys. The increase may be ascribed to an accumulation of their appropriate materials in the blood. Even if the tissues which may be said to be the most independent and self-sustaining be deprived of the requisite amount of nutriment, they suffer in development, and are modified by abnormal substances in the blood. These substances single out particular organs, and certain parts of them, exerting their deleterious influences upon them in such a manner as to show that they do possess an elective affinity. From what has been said it will be apparent in order to proper nutrition the blood must contain the requisite quantity of its constituents in the requisite state of purity. We have now only to consider its relations to the tissues.

The demand for nutrition arises primarily from a tendency of the organism to simple increase or growth. This is most strikingly illustrated in the multiplication of the first embryonic cell, by the simple process of "duplicative subdivision," whereby a multitude of cells are evolved each one of which resembles the original in all essential points. But after the different parts of this embryonic mass have taken upon themselves their respective modes of development, so as to generate a diversity of tissues and organs, each continues to increase after its own plan, thus the child becomes the adult with comparatively little change but that of growth.

An excess of growth after the normal plan of the tissue or organ is called hypertrophy, whilst a diminution without any marked degeneration or alteration of it is called atrophy. Growth is not confined to the period of the increase of the body, but may occur at any time, consequent on increased activity in any set of muscles. There is an increase taking place in the active organs at all times, even to the end of life, but this may be overbalanced by retrograde metamorphosis, and instead of increase there may be actual diminution. The evolution of the complete organism does not consist in mere growth, for it would produce nothing but an enormous aggregation of cells. In addition to increase there must be development, that is a passage to a higher condition both of form and structure, which fits them for their functions in the animal economy. Thus the development of tissue consists in the change of a simple mass of cells into another form, as in the production of the dentine from the cellular substance of the tooth pulp. Thus again the developmental change is seen in the passage of an entire organ from a lower to a higher condition, by the evolution of new parts, or by a change in those already existing, even though the change in its texture should be little more than that of simple growth, as in the casé of the heart. It has at first but one cavity, then by the formation of a partition it obtains two and finally by the same process four cavities. It is in the early part of embryonic life that these developmental processes are most remarkably displayed, it is now that the transformation of simple cells into tissues of various kinds occur, now a "nisus" originates in each part whereby the production of the same tissue becomes a simple act of growth. It is now that the marking out of each organ takes place, so that its further growth is a simple filling up of the plan now made. Different organs take different measures of time for their growth, and when once the animal has reached its complete growth, every subsequent change is one rather of degeneration than development, retrogression than of advance.

The difference between growth and development is best shown where there is a partial or complete arrest of one, without a corresponding impairment of the other. Thus, a dwarf may present perfect development, the deficiency being only in growth. On the other hand a child may have attained the usual size at birth, and yet possess a heart with but one cavity. This would be a case of arrested development.

The demand for nutrition does not arise merely from the exercise of the formative capacity, but also from the decay and degeneration, which is constantly taking place, which if not counterbalanced would result in the complete disintegration of the organism. As each component cell of the body has to a certain degree an independent life of its own, so has it also a limited duration, and this duration corresponds with its functional activity. This may be seen in the blood corpuscles, in the epithelial cells of many of the secreting vessels, and also in the muscular, nervous and osseous tissues. Thus it happens, that during the whole period of active life, the demand for nutrition is created by every exertion of the vital powers, especially by the evolution of the muscular and nervous forces. The production and application of these may be considered as the great aim and end of the human organism so far as the individual is concerned. In the early stages and in that of youth, the productive power is very great, but in the mature man there is an increase in the exercise of the musculo-nervous power, and a decrease in the productive power so that the productive power is only enough to make good the loss occasioned by the retrograde metamorphosis. While in advanced life the loss becomes greater than the supply, though the size of the person may remain about the same, or even become greater, on account of an increase in fat.

The performance of the function of nutrition does not alone depend on a due supply of pure and well elaborated blood, but also upon the normal condition of the part to be nourished, and especially upon the possession of a right measure of formative power, in virtue of which the newly formed tissues are generated in the likeness as well as in the place of those, which have become effete.

Varying activity of the nutritive process, without any change in the character of the nutritive processes, there may be considerable variation in their degree of activity, and this may take place in the whole organism, or be confined to particular parts, most generally the latter. These variations may be so considerable as to constitute disease, though there are some that occur as the regular effect of physiological phenomena. Of the latter I may mention as a common example the growth and maturity of the ovule, and the growth of the womb after conception.

The abnormal nutritive change which causes hypertrophy, has for its source one or more of three causes, viz: a departure from the right composition of the blood, of the due supply of such blood, and of a proper formative capacity of the blood. Some cases are to be attributed to the greater afflux of blood to a part, as when in the vicinity of an ulcer the hair grows more rapidly and longer. Perhaps the increased thickness of the skin in parts exposed to friction is due to the same cause.

The greater number of cases, however, may be referred to preternatural formative capacity in the tissue itself, and this may be either congenital or acquired. (Ranke & Capenter.) In case of greatly enlarged limbs, or of the fingers and toes, we can generally say that they are congenitally enlarged. But when the muscular coat of the bladder is enlarged, it is generally owing to stricture or stone in the bladder. This enlargement is beyond doubt acquired, caused by the existence of these obstructions to the elimination of the urine. The exercise of the muscles causes an enlargement of them, as may be observed in blacksmith's arms. I think it hardly necessary to enter into an account of the manner in which these enlargements take place.

Tumors may be considered as species of hypertrophy, for they may be composed of tissues normal to a part. The tumor follows in its growth no natural shape or construction the longer it continues to increase, the greater its deformity (Paget.)

Carpenter also ascribes such monstrosities as double head, two limbs, supernumerary fingers, toes, etc., not to two germs, but to one possessing extraordinary formative capacity, thus evolving the supernumerary parts.

The state of atrophy is the reverse of that of hypertrophy. Here there is a reduction in the formative capacity. The tissue formation is not equal to the decay and waste, so there is a diminution in the size, and a deterioration in the quality and condition of the part generally. Atrophy may be either general or local, of local atrophy we have an example in the rapid decrease of the uterus after parturition. General atrophy may be seen in certain wasting conditions of the system, arising from continuous exertions either of the body or of the mind, we may look for the causes of atrophy in the condition and supply of the blood, and in the formative capacity of the tissues themselves. The atrophy depending upon an insufficient supply of blood may be either general or local. General atrophy or emaciation is a natural consequence of a deficiency of food, but it may depend upon an imperfect performance of the assimilating processes, or upon an unusual activity of the process of retrograde metamorphosis whereby the nutritive materials are not assimilated, but decomposed into excrementitious products, as perhaps is the case in diabetes. Examples of the atrophy of particular tissues can easily be given, but I shall only mention that of the adipose tissue. The substance composing this tissue, fat when there is no surplus in the blood to serve for its nutrition, is drawn into the circulation and there used perhaps to keep up the animal heat, at the same time the muscular tissues may actually increase in size. The most common cause of atrophy may be referred to deficient formative capacity in the tissues themselves, I believe that fatty degeneration, the form in which degeneration generally manifests itself, is more common than simple wasting (Ranke & Carpenter) but it attracts less notice than wasting, because the ordinary size and bulk is maintained by the organs. It does not seem to arise from a lack of exercise or activity of function, because it most frequently appears in those organs that are in constant activity, as the heart, lungs, liver, etc.,

Nutrition may be well studied in the reparatory processes, because here it takes place with unusual rapidity and activity.

